# The Association of M235T Genetic Polymorphism in *Angiotensinogen* Gene and Other Non-Genetic Factors with Essential Hypertension among Jordanian Patients

**DOI:** 10.3390/jpm14030273

**Published:** 2024-02-29

**Authors:** Hussein Alhawari, Yazun Jarrar, Malek Zihlif, Ayman Wahbeh, Sameeha Alshelleh, Ruba Alhabahbeh, Dalia Abdelrazaq

**Affiliations:** 1 Department of Internal Medicine, School of Medicine, The University of Jordan, Amman 11942, Jordan; h.alhawari@ju.edu.jo (H.A.); a.wahbeh@ju.edu.jo (A.W.); s.shelleh@ju.edu.jo (S.A.); 2 Department of Basic Medical Sciences, Faculty of Medicine, Al-Balqa Applied University, Al-Salt 19117, Jordan; 3 Department of Pharmacology, School of Medicine, The University of Jordan, Amman 11942, Jordan; m.zihlif@ju.edu.jo (M.Z.); daliamuhmod@gmail.com (D.A.); 4 Department of Community Medicine and Public Health, School of Medicine, The University of Jordan, Amman 11942, Jordan; r.alhabahbeh@ju.edu.jo

**Keywords:** *AGT M235T* polymorphism, essential hypertension, genetic investigation, Jordan

## Abstract

Background: Hypertension, characterized by elevated pressure, poses a significant health risk. Recent studies in Jordan highlight high hypertension rates, emphasizing the need for genetic investigations to comprehend essential hypertension determinants. The *AGT* gene, part of the Renin Angiotensin System, is linked to blood pressure regulation. Limited information exists on the frequency of this polymorphism among Jordanian hypertensive patients. Aims: This study explores the association between the *AGT M235T* polymorphism and essential hypertension in Jordan. Methods: A cross-sectional study with 435 participants (199 hypertensive, 236 non-hypertensive) was conducted at the University of Jordan Hospital. Blood pressure was measured, and genetic analysis of the *AGT M235T* polymorphism was completed using the PCR-RFLP technique. Chi-square and *t*-tests were used for comparisons using SPSS software. Results: Hypertensive patients exhibited significantly higher weight, BMI, and blood pressure. Genotyping results showed no significant difference (*p* > 0.05, *Chi-square*) in *AGT M235T* polymorphism distribution between control and patient groups. In addition, allele frequencies showed comparable patterns (*p* > 0.05, *Chi-square*). All genotype frequencies showed no deviation from the Hardy–Weinberg equation (*p* > 0.05, *Chi-square*). Conclusions: The *AGT M235T* genetic polymorphism is not more prevalent among hypertensive patients in Jordan, although the average weight and BMI among hypertensive patients is higher than the non-hypertensive participants. Obesity can be addressed as a potential risk factor for essential hypertension in Jordan. In addition, it is recommended to find out the influence of the *AGT M235T* genetic polymorphism on the response of antihypertensive drugs among hypertensive patients in Jordan.

## 1. Introduction

Blood pressure, defined as the force exerted by the circulation of blood against arterial walls, is a major physiological parameter integral to cardiovascular health. Blood pressure is expressed as two values: systolic, representing pressure during cardiac contraction, and diastolic, indicative of pressure in between contractions [1]. Hypertension, a condition characterized by elevated blood pressure levels, is clinically diagnosed when the systolic blood pressure (SBP) exceeds 140 mm Hg and/or the diastolic blood pressure (DBP) surpasses 90 mm Hg [2]. Hypertension is one of the major cardiovascular diseases with complications and considered one of the major causes of mortality across the world [2]. 

The prevalence of essential hypertension is high among different developed and developing countries [1,3]. Recent studies conducted in Jordan have showed that the rate of hypertension is 41.4% for men, while it is 28.3% for women [3,4]. Accordingly, it is expected that the number of hypertensive cases in Jordan is increasing, resulting in an elevated risk of coronary heart disease.

Essential hypertension is influenced by a complex of genetic and environmental factors, such as the type of food, and the presence of other diseases, such as kidney disease [1,2]. Genetic determinants play a substantial role, contributing to approximately 30% of the variability observed in blood pressure levels [5]. 

The Renin Angiotensin System (RAS), a key regulatory system governing blood pressure homeostasis, fluid balance, and electrolyte metabolism, holds particular significance in this context [6]. Dysfunction in the RAS causes cardiovascular diseases, including hypertension and heart failure [6]. Angiotensinogen (AGT) is a key component of the RAS. It is a naturally occurring substrate that the liver synthesizes [7]. The angiotensinogen protein is encoded by the *AGT* gene. Genetic studies have highlighted the *AGT* gene and its relationship with the cardiovascular diseases [8,9,10].

The *AGT* gene, located on chromosome 1q42-43, comprises four introns and five exons, spanning a genomic sequence of 13 kb. With a molecular weight of approximately 50,000 Da, the mature form of *AGT* consists of 452 amino acid residues [11]. Several polymorphisms within the *AGT* gene have been identified, with particular emphasis on the *M235T* polymorphism [8,9,10]. This genetic variant involves a single-base pair substitution of thymine with cytosine at nucleotide 704 (T704C), resulting in the replacement of methionine (M allele) by threonine (T allele) at position 235 [11]. 

To the best of our knowledge, there is a lack of information regarding the frequency of *AGT M235T* genetic polymorphism among hypertensive patients in Jordan. Accordingly, this study aims to investigate the association between the *AGT M235T* polymorphism and essential hypertension within the Jordanian population. This study hypothesized that the *AGT M235T* polymorphism is more frequent among hypertensive patients in Jordan. This research aims to find out whether the *AGT M235T* polymorphism can be served as a genetic biomarker for the development of essential hypertension among individuals in Jordan. The results of this research can increase our understanding of the genetic background among Jordanian population and its association with the common diseases, including essential hypertension. 

## 2. Materials and Methods

### 2.1. The Study Design

This study is a cross-sectional study that was undertaken with an enrollment of 435 participants, from both men and women. Among them, 199 individuals were diagnosed with essential hypertension, while the remaining 236 were considered the control non-hypertensive group. This study was completed during October 2022 to December 2023. 

The age of the participants in this study ranged between 25 and 65 years. Blood pressure measurements were obtained using a sphygmomanometer, with readings taken at least three times at five-minute intervals. During measurements, subjects were seated in a chair with back support, their feet on the ground, and their arms comfortably resting on a table at heart level. The control group consisted of individuals with a systolic blood pressure <140 mm Hg and a diastolic blood pressure <90 mm Hg, while the essential hypertension group had a systolic blood pressure >140 mm Hg and/or a diastolic blood pressure >90 mm Hg, according to the American Heart Association guideline of hypertension [12]. The diagnosis of hypertension was completed by specialists at the University of Jordan Hospital. 

Exclusion criteria ensured that participants had no history of diabetes mellitus, secondary hypertension, renal failure, coronary heart disease, liver dysfunction, or thyroid disease.

The participants in this study were Arabs, the major ethnic group in Jordan. We identified the ethnicity of the participants through a direct question to the participant and a revision of the family name that indicates the ethnicity of the volunteer.

Each participant provided an informed consent before the participation in this study. In addition, the ethical committee of the University of Jordan Hospital approved the protocol of this study with a reference of 10/2022/24730. The institutional review board of the University of Jordan Hospital reviewed the protocol of this study and confirmed that it followed the declaration of Helsinki. 

The demographic data of the participants were obtained from the computer records in the University of Jordan Hospital, in addition to the direct question asked of the participant. 

Given the patients have high blood pressure without other chronic diseases, and with about 2000 patients coming to the University of Jordan Hospital every year, it was decided on a sample size of 236 patients. This choice was made with a statistical power (1 − β) of 0.8, a margin of error of 5%, and a confidence level of 95%. The calculation of the sample size of this study was done through the Raosoft online calculator (http://www.raosoft.com/samplesize.html, accessed on 1 October 2022).

### 2.2. Genotyping

Blood samples, ranging from three to five ml, were collected in Ethylenediaminetetraacetic acid (EDTA) tubes for subsequent genetic analysis. The tubes were labeled with serial numbers referring to the participants rather than their names. The extraction of genomic DNA from peripheral blood samples was conducted using the commercially available Wizard Genomic DNA Purification Kit (Promega, Madison, WI, USA), in accordance with the manufacturer’s recommended protocol. Specifically, genomic DNA was isolated from 300 µL blood leukocytes obtained from the samples. The leukocytes were exposed to 1ml cell lysis and 300 µL nucleu-lysis, then the 100 µL protein precipitation solutions. Then, the genomic DNA was precipitated using 500 µL isopropyl alcohol and it was washed using 500 µL 70% ethanol. Lastly, the DNA pellets were rehydrated using nuclease-free water (Promega, USA). The concentration of the extracted DNA was determined using the Nanodrop instrument Quawell DNA/Protein Analyzer (Thermo Fisher Scientific, Sunnyvale, CA, USA). The purity of all DNA samples was indicated by the 280/260 ratio. Only samples with a ratio of 1.8 ± 0.1 were accepted for further genetic analysis. Following the concentration assessment, the samples underwent a dilution process, utilizing nuclease-free water to achieve a standardized final concentration of 200 ng/μL. 

**Figure 1:**
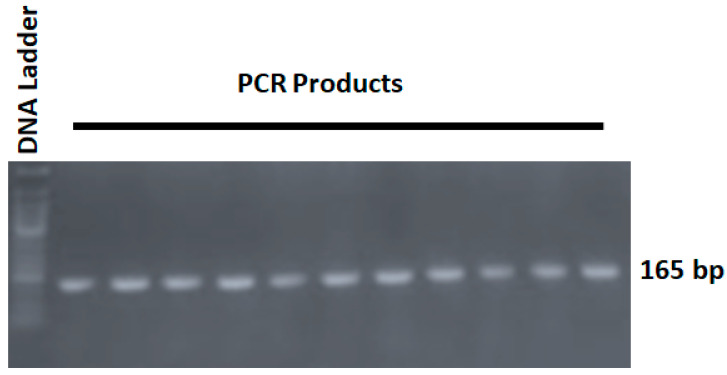
Gel electrophoresis of the PCR products of the *AGT* gene containing the *AGT T704C* genetic polymorphism. The first lane represents the 50-bp DNA ladder. The remaining lanes are random representative PCR products of the participants in this study. The PCR bands were visualized using Redsafe dye (iNTRON, Seongnam-si, Republic of Korea).

Genetic analysis involved the amplification of genomic DNA by polymerase chain reaction (PCR) (Bio-Rad, Hercules, CA, USA). Primer pairs (SNP Biotechnology R&D Ltd., Ankara, Turkey) were selected to amplify the DNA fragment containing the *AGT* gene (T704C, rs699) polymorphism, as was published previously [13]. The sequence of the forward primer is 5′-CCG TTT GTG CAG GGC CTG GCT CTC T-3′, while the reverse primer is 5′-CAG GGT GCT GTC CAC ACT GGA CCC C-3′. The commercial PCR master mix (Solis Biodine, Tartu, Estonia) was prepared, which contained the DNA polymerase, a mixture of deoxynucleotide triphosphate, Mgcl_2_, nuclease free water, and the buffer. The total volume of the PCR reaction was 20 μL, containing 4 µL of the 5× PCR master mix, 10 pmole of forward and reverse primers, and 200 ng of gDNA; the volume was completed by the nuclease free water. The protocol of the PCR reaction included initial denaturation at 94 °C for 5 min, followed by a PCR cycle of denaturation at 94 °C for 30 s, annealing at 58 °C (the primers’ annealing temperature) for 30 s, and extension at 72 °C for 45 s, repeated for 35 cycles. The final extension step was performed at 72 °C for 10 min. The confirmation of the PCR reaction involved the gel-electrophoresis technique conducted as follows: 5 µL of the PCR product was loaded onto a 1.5% agarose gel (Life Technologies, Carlsbad, CA, USA). Subsequently, an electrical current of 125 A was applied for a duration of 20 min using the Bio-Rad Power PacTM Basic system (Bio-Rad, Hercules, CA, USA). Subsequently, the gel was stained with Redsafe dye (iNTRON, Seongnam-si, Republic of Korea). To determine the size of the PCR products, a standard-size 100 bp DNA loading ladder (Promega, Madison, WI, USA) was used as a reference. The visualization of the DNA bands was achieved using a benchtop UV transilluminator (Bio Doc-ITTM, Madras, India). The size of the PCR product was 165 base pairs (bp), as represented in Figure 1.

**Figure 2:**
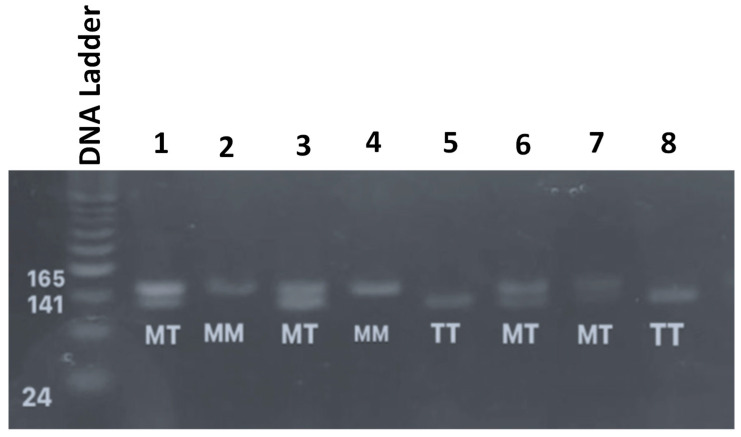
Genotyping of the *AGT T704C* genetic variants. The first lane is the DNA ladder. Lanes 1, 3 and 6 represent the heterozygous genotype, lanes 2 and 4 represent the homozygous genotype and lanes 5 and 8 represent the wild *AGT T704C* genotype.

For the *AGT* gene (T704C), PCR-RFLP (Restriction Fragment Length Polymorphism) was conducted by digesting the PCR product with one unit of *Psyl (Tth 111 I)* (ThermoFisher Scientific, Waltham, MA, USA) restriction enzyme for 8 h. The restriction enzyme was denatured using a high heat (65 °C) for 5 min. Then, the products after digestion with the restriction enzyme were separated using 2.5% agarose gel electrophoresis (Bio-Rad, Power PacTM Basic, Hercules, CA, USA), using an electrical current of 100 A for a duration of 40 min. Visualization of the DNA bands was achieved using a benchtop UV transilluminator (Bio Doc-ITTM, Madras, India). Figure 2 represents the three *AGT T704C* genotypes (wild, heterozygous, and homozygous) after gel electrophoresis. 

### 2.3. Statistical Analysis

The statistical analysis employed various tests to scrutinize the *AGT* gene *(T704C)* genotypes and associated data. The *Chi-square (χ*^2^*)* test assessed the distribution of *AGT (T704C)* genotypes between the control and hypertensive groups. Additionally, the genotypic frequencies underwent scrutiny for adherence to the Hardy–Weinberg equilibrium using the χ^2^ test. Comparative analysis of *AGT (T704C)* genotypes among Jordanians and frequencies observed in other ethnic groups was completed through the χ^2^ test. Continuous data, comparing variables between control and hypertensive groups, underwent evaluation using the one-tailed Student *t*-test. The statistical analyses were performed using SPSS software, version 26, by IBM, Armonk, NY, USA. The significance threshold was set at a *p* value below 0.05.

## 3. Results

### 3.1. Demographic Data

Table 1 shows the anthropometric data of both groups. In the control group, participants exhibit an average age of 34.69 ± 11.79 years. The mean height is 166.56 ± 11.01 cm, and the average weight is 77.38 ± 16.62 kg. The Body Mass Index (BMI) has a mean of 27.51, suggesting a moderate level of obesity. SBP averages 115.64 ± 6.14 mm Hg, while DBP averages 76.06 ± 11.92 mm Hg.

Contrastingly, the hypertensive patient group demonstrates a higher average age of 45.48 ± 9.99 years. The mean height is 168.37 ± 10.11 cm, and the average weight is 88.71 ± 18.39 kg. The BMI for this group is notably higher, with a mean of 31.30, indicating a higher prevalence of obesity. SBP for hypertensive patients averages 160.84 ± 12.96 mm Hg, and DBP averages 100.47 ± 7.67 mm Hg.

The statistical analysis reveals significant differences (*p* < 0.05, *t-*test) between the two groups, with hypertensive patients displaying higher average weight, BMI, and both systolic and diastolic blood pressure compared to individuals in the control group.

### 3.2. Genotyping Results 

The genotype results for the *AGT* gene (T704C) in both the control and patient groups are presented in Table 2. The distribution of genotypes, including wild-type (wild), heterozygous (Hetero), and homozygous mutant (M), was assessed. In the control group, the wild-type genotype constitutes 32.16%, the heterozygous genotype accounts for 50.75%, and the homozygous mutant genotype comprises 17.08%. Conversely, in the patient group, the frequencies are 27.54%, 52.54%, and 19.92% for the wild-type, heterozygous, and homozygous mutant genotypes, respectively. Statistical analysis indicates no significant (*p* > 0.05, *Chi-square*) difference in the genotype distribution between the control and patient groups. All frequencies of the *AGT M235T* genotypes showed no deviation from within the Hardy–Weinberg equation (*p* > 0.05, *Chi-square*). Furthermore, the control group exhibits a wild-type allele frequency of 57.54% and a mutant allele frequency of 42.46%. Similarly, the patient group shows a wild-type allele frequency of 53.81% and a mutant allele frequency of 46.18% (Table 3). 

### 3.3. Inter-Ethnic Variation in the Frequency of AGT M235T Genotype 

The inter-ethnic variation in the distribution of the *AGT M235T* genotype is evident across the diverse racial groups, as presented in Table 4. Chinese people have the highest frequency (54.6%) of the *AGT M235T* genotype, which is significantly (*p* < 0.05) different from other ethnic groups. In addition, the Indian population has a significant (*p* < 0.05) number of people (31.5%) with the *AGT M235T* genotype. On the other hand, the Turkish and Greek populations have significantly fewer people (2.4%, and 7%, respectively) carrying the *AGT M235T* genotype than the Jordanian population. Lastly, there is no significant difference in the frequency of the *AGT M235T* genotype between the Jordanian and Caucasian German populations. 

## 4. Discussion

Essential hypertension is a common health concern in Jordan [3,4]. Despite its widespread occurrence, there’s been a notable lack of research investigating the relationship between genetic variations and this condition in the Jordanian population. Consequently, our knowledge about the specific genetic factors linked to hypertension remains largely incomplete in this demographic. Recognizing this gap, there is a need for extensive research to discover the genetic landscape associated with hypertension in Jordan. Such research helps us to provide valuable insights into the various factors contributing to this prevalent health issue, offering a more comprehensive understanding. In this context, our current study investigated the connection between *AGT gene (T704C)* polymorphism and specific non-genetic factors with high blood pressure among individuals in Jordan. It is crucial to emphasize that, as far as our knowledge extends, no prior studies in Jordan have undertaken the task of exploring the genotyping of the *AGT gene (T704C)* among individuals with hypertension. The outcomes of this study have the potential to enhance our understanding, at least partially, about essential hypertension, a highly prevalent cardiovascular ailment in Jordan [3,4]. This research contributes to the growing body of knowledge surrounding the genetic and non-genetic factors influencing high blood pressure in the Jordanian context.

In the control group, the calculated BMI of 27.51 places the control group within the range of moderate obesity [19]. The blood pressure parameters, with an average SBP of 115.64 mm Hg and DBP of 76.06 mm Hg, fall within the normotensive range among Jordanians [12]. Contrastingly, the hypertensive patient group among Jordanians exhibits a notably higher average weight of 88.71 kg contributing to a significantly (*p* > 0.05, *t-*test) higher BMI of 31.30, indicating a higher prevalence of obesity within this Jordanian hypertensive group [19]. The blood pressure parameters reveal significantly elevated levels, with an average SBP of 160.84 mm Hg and an average DBP of 100.47 mm Hg, placing the hypertensive patient group firmly within the hypertensive range among Jordanian patients [12]. The hypertensive patients, on average, exhibit a significantly (*p* > 0.05, *t-*test) higher weight and BMI compared to the control group, providing valuable insights into hypertension patterns among Jordanian individuals. Accordingly, obesity can be considered as a risk factor of essential hypertension among Jordanian patients. This finding is in line with what was found previously, that obesity is a risk factor of essential hypertension [20,21]. It was reported that obesity increases the blood volume through the adipose tissue producing various bioactive substances and hormones, such as leptin, which can lead to an increase in blood volume [22,23]. In addition, obesity can increase the blood pressure through the activation of the Renin-Angiotensin-Aldosterone System by hormones and cytokines which are produced by the adipose tissue [24,25]. Accordingly, it is recommended to inform the hypertensive patients to reduce their body weight, especially since the prevalence of obesity is high among the Jordanian population. Some studies showed that obesity also linked to other prevalent diseases in Jordan, such as diabetes mellitus [26]. In addition, some studies showed genetic variants associated with obesity also linked with chronic diseases [27]. 

The genotyping results show the distribution of wild-type, heterozygous, and homozygous mutant genotypes in both the control and hypertensive patient groups of Jordanians. However, the statistical analysis indicates no significant difference (*p* > 0.05, *Chi square-test*) in the genotype, in addition to the allele distribution between the two groups. These findings may indicate that the *AGT gene (T704C)* genetic variant is not associated with the susceptibility of essential hypertension among Jordanian patients. This finding agrees what was reported in the study of Zeng et al. (2010) among Chinese patients [28], Glavnik et al. (2007) [18] among Caucasian patients, and Mohana et al. (2012) [14] among Indian hypertensive patients. However, it disagrees what was reported by Abdullaeva et al. (2023) among Uzbek patients [29], Agachan et al. (2003) [16] among Turkish patients, and Kolovou et al. (2015) [9] among Greece hypertensive patients, that the frequency of mutant *AGT gene (T704C)* polymorphism is higher among the hypertensive patients. Accordingly, there are still controversial results regarding the association of *AGT M235T* with essential hypertension. Further clinical studies are needed to confirm the association of *AGT M235T* with the essential hypertension through a multi-central and multi-ethnic study. 

The Jordanian population is often categorized as Caucasian, yet it possesses a distinct genetic makeup due to historical interactions with Africans and Asians [30]. This unique blend results in a one-of-a-kind genetic background among Jordanians. Consequently, the frequency of certain functional genetic variants may vary significantly compared to what is reported in other Caucasian ethnic populations [31]. This diversity highlights the importance of exploring genetic variations specific to the Jordanian context. We found in the present study that there is an inter-ethnic variation in the frequency of the *AGT M235T* genotype. We compared the frequency of the *AGT M235T* genotype found in this study with what was reported in the previous studies and found that the frequency of the *AGT M235T* genotype is significantly (*p* < 0.05, *Chi square-test*) different than Asian Chinese and Indians [14], Turkish [16], and Greeks [9], while it is incomparable (*p* > 0.05, *Chi square-test*) to frequencies with the German population [15]. This inter-ethnic variation in the frequency of the *AGT M235T* genotype may explain, at least partly, the inter-ethnic variation in the predisposition to the diseases, including essential hypertension [32].

Pharmacogenetic studies have explored how genetic variations might influence the response to antihypertensive drugs that target RAAS [33]. Angiotensin-converting enzyme (ACE) inhibitors and angiotensin II receptor blockers (ARBs) are common medications that interfere with RAAS to lower blood pressure. In individuals with the *AGT M235T* polymorphism, variations in angiotensinogen levels could potentially affect the efficacy of these drugs. As a result, pharmacological interventions targeting angiotensin II production or action may have varying effects based on the individual’s genetic profile. Understanding the pharmacogenetic implications of the *AGT M235T* variant may help in the personalized medicine approaches in hypertension management. Accordingly, it is recommended to investigate the influence of the *AGT M235T* polymorphism on the response of antihypertensive medications among Jordanian hypertensive patients.

While this study successfully achieved its goals, it does have certain limitations. Firstly, it focused on a single functional genetic polymorphism in the *AGT* gene. To enhance our understanding, it is advisable to extend the investigation by sequencing the entire *AGT* gene among hypertensive patients in Jordan. This broader approach may unveil rare functional *AGT* genetic variants linked to hypertension. Secondly, the study included participants solely from the major ethnic group in Jordan, the Arabs, neglecting minority groups like Circassians, Kurds, and Chechens. This omission limits the generalizability of the findings to the entire Jordanian population. Lastly, participants were recruited exclusively from a hospital in the capital city, excluding those residing in distant areas or small villages. Recognizing these limitations, future studies should aim for more comprehensive inclusion and representation to capture a more accurate picture of the diverse Jordanian population.

## 5. Conclusions

In conclusion, our study at the University of Jordan Hospital investigated the association between the *AGT M235T* polymorphism and essential hypertension in Jordan. Despite revealing a higher average weight and BMI among hypertensive patients, the genotyping results demonstrated no significant association with the *AGT M235T* polymorphism. The study underscores the complex interaction of genetic and non-genetic factors contributing to hypertension in Jordan. While the investigation focused on a single polymorphism, future research should explore the entire *AGT* gene and include a more diverse participant pool, considering the unique genetic makeup of the Jordanian population. The findings also emphasize the importance of addressing obesity as a potential risk factor for essential hypertension in Jordan.

## Figures and Tables

**Table 1:** The anthropometric data of the participants.

Participants	Number of Samples	Gender (Frequency, %)	Age (Mean ± S.D.)	Height (Mean ± S.D.)	Weight (Mean ± S.D.)	BMI (Mean ± S.D.)	Smocking (Frequency, %)	SBP (Mean ± S.D.)	DBP (Mean ± S.D.)
Control	199	Male = 77(39%) Female = 122 (61%)	34.7 ± 11.8	166.6 ± 11.0	77.4 ± 16.6	27.5 ±5.7	Smokers = 65 (32%) non-smokers = 134 (68%)	115.6 ± 6.1	76.1 ± 12.0
Patients	234	Male = 120 (51%) Female = 116 (49%)#	45.48 ± 9.99	168.37 ± 10.11	88.71 ± 18.39 *	31.30 ± 5.69 *	Smokers = 78 (33%) non-smokers = 158 (67%)	160.84 ± 12.96 *	100.47 ± 7.67 *

The anthropometric data were collected from the computer records in the University of Jordan Hospital. “*” indicates statistical significance using *t-*test, *p* < 0.05. BMI is the abbreviation of body mass index, SBP is the abbreviation of systolic blood pressure, and DBP is the abbreviation of diastolic blood pressure.

**Table 2:** Frequencies of the *AGT M235T* genotypes among the participants.

Genotype	Wild (MM)	Heterozygous (MT)	Homozygous (TT)
Control	64 (32%)	101 (51%)	34 (17%)
Patients	65 (28%)	124 (53%)	47 (20%) ^@^

^@^ The statistical analysis was completed using *Chi-square* test. No statistical significance in the comparison between genotype frequencies of both groups. All genotype frequencies are within Hardy–Weinberg equation (*p* > 0.05, *Chi-square*).

**Table 3:** Frequencies of the *AGT M235T* alleles among the participants.

Allele	Wild (M)	Variant (T)
Control	229 (58%)	169 (42%)
Patient	254 (54%)	218 (46%) ^@^

^@^ The statistical analysis was completed using *Chi-square* test. There was no statistical significance in the comparison between genotype frequencies of both groups.

**Table 4:** The frequency of *AGT M235T* genotype among different ethnic populations.

Ethnic Population	Frequency of the *AGT M235T* Genotype	Total Number of the Non-Patients	Reference
Greece	7.2% *	83	[9]
India	31.5% *	200	[14]
Germany	14.5%	124	[15]
Turkish	2.4% *	74	[16]
Chinese	54.6% *	185	[17]
Caucasians	21.1%	404	[18]
Jordan	17%	199	This study

“*” indicates statistical significance using *Chi square-test*, *p* < 0.05.

## Data Availability

Data are available with the corresponding author upon request.
